# Magnetic solid-phase extraction of caffeine from surface water samples with a micro–meso porous activated carbon/Fe_3_O_4_ nanocomposite prior to its determination by GC-MS†

**DOI:** 10.1039/d1ra01564h

**Published:** 2021-05-28

**Authors:** Natalia Manousi, Eleni A. Deliyanni, Erwin Rosenberg, George A. Zachariadis

**Affiliations:** Laboratory of Analytical Chemistry, Department of Chemistry, Aristotle University of Thessaloniki Thessaloniki 54124 Greece; Laboratory of Chemical and Environmental Technology, Department of Chemistry, Aristotle University of Thessaloniki Thessaloniki 54124 Greece; Institute of Chemical Technologies and Analytics, Vienna University of Technology Getreidemarkt 9/164 1060 Vienna Austria erwin.rosenberg@tuwien.ac.at

## Abstract

A novel micro–meso porous activated carbon/Fe_3_O_4_ (Bm) composite was synthesized from the active charcoal precursor BAX-1500 and used in the magnetic solid-phase extraction (MSPE) of caffeine prior to its determination by gas chromatography-mass spectrometry (GC-MS). The main factors affecting the extraction and desorption steps of the MSPE procedure were investigated and optimized. These factors include extraction time, sorbent mass and salt addition for the adsorption step and type of eluent, desorption time and volume of desorption solution for the desorption step. Under optimum conditions, the absolute extraction recovery was found to be 91.1% and good linearity was observed in the investigated concentration range of 0.6–12.5 ng mL^−1^ (*R*^2^ = 0.9997). The limit of detection was 0.18 ng mL^−1^ and the limit of quantification was 0.60 ng mL^−1^. The method was successfully applied to the analysis of surface water samples. The proposed MSPE method is simple, rapid, sensitive and environmentally friendly.

## Abstract 2

A novel magnetic solid phase material based on a micro–meso porous activated carbon/Fe_3_O_4_ nanocomposite was used to extract caffeine from surface water samples. The method is efficient and rapid, and has minimum solvent consumption.

## Graphical abstract

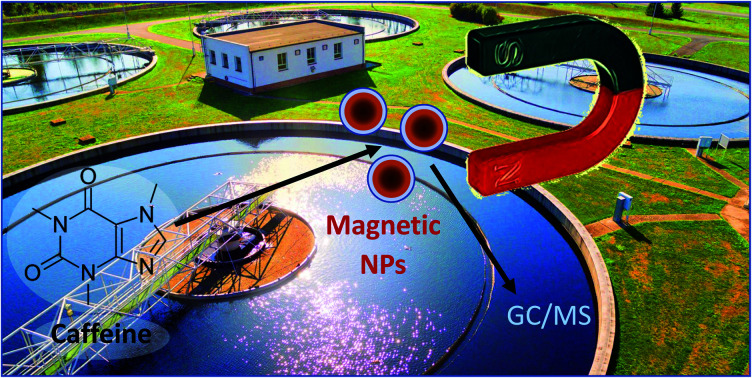

## Introduction

Magnetic solid-phase extraction (MSPE) is a form of dispersive solid-phase extraction in which a magnetic sorbent is directly added into an aqueous sample.^1,2^ Due to the intensive contact of the sorbent and the target analytes during the stirring-supported extraction step, extraction efficiency significantly increases and mass transfer of analytes to the active sites of the sorbent is facilitated.^3^ After the extraction of the target analytes, an external magnetic field is applied to separate the magnetic sorbent from the solution and elution with an appropriate solvent is performed. MSPE is a rapid, convenient and efficient sample preparation technique that has been successfully employed for the analysis of environmental, biological and food matrices.^1,4^

Caffeine is an alkaloid that is constituent of a variety of foods (chocolate, pastries *etc.*) and beverages (coffee, tea, soft drinks). Caffeine is a useful potential chemical marker for the estimation of domestic human wastewater contamination. Therefore, the determination of caffeine in surface water samples is critical to evaluate the impact of anthropogenic activity for waste water contamination of surface water. Since the concentration of caffeine in surface water may be low, preconcentration is considered a necessary step in sample preparation.^5,6^

Caffeine is usually determined by high performance liquid chromatography (HPLC) combined with a variety of detection systems including ultraviolet (UV),^7^ diode array detection (DAD)^8^ and mass spectrometry (MS).^9^ However, HPLC analysis may involve the use of hazardous solvents as mobile phases, plus the inconvenience of buffer preparation for pH adjustment. Gas chromatography (GC) can be applied to avoid these time- and cost-consuming steps and is also environmentally more benign.^10^ Therefore gas chromatography combined with a flame ionization detector (FID)^10^ or mass spectrometer^11^ has been successfully employed for the determination of caffeine in a variety of matrices. Until now, solid-phase extraction (SPE)^12^ and liquid–liquid extraction (LLE)^13^ are the two major conventional sample preparation techniques that are used for the extraction of caffeine from a plethora of samples. However, these techniques exhibit some fundamental limitations since they comprise of time-consuming steps and they require the consumption of high quantities of sample and organic solvents.^14^ Compared to the conventional methods for the determination of caffeine, MSPE is rapid and a convenient choice.

Various magnetic sorbents have been employed for the magnetic solid-phase extraction including activated carbon,^15^ metal–organic frameworks,^16^ molecularly imprinted polymers^17^ and graphene oxide.^4^ Activated carbon exhibits high surface area, a wide range of surface functional groups and internal microporosity, therefore it has proved to be an effective sorbent that can be used for the separation and purification of gaseous and liquid phase mixtures.^15,18^ Magnetic activated carbons are composite materials that consist of activated carbon and magnetic labels, including magnetite (Fe_3_O_4_) and maghemite (γ-Fe_2_O_3_).^15,19^ Magnetic activated carbon has been successfully employed for the MSPE of various analytes such as bisphenol A,^15^ tartrazine,^20^ phenylurea herbicides^21^ and parabens.^22^

In this work, a micro–meso porous activated carbon/Fe_3_O_4_ (Bm) nanocomposite was prepared from the active charcoal precursor BAX-1500 and investigated for the magnetic solid-phase extraction of caffeine prior to their determination by gas chromatography-mass spectrometry. The main experimental parameters that influence the adsorption and desorption steps were optimized and the developed method was validated. The adsorption mechanism of caffeine onto the nanocomposite was evaluated. The novel sorbent was successfully applied, for the first time, for the MSPE of caffeine from surface water samples prior to its determination by GC-MS.

## Experimental

### Chemicals and reagents

All chemical used during method development were of analytical grade, unless stated otherwise. Caffeine was purchased from Sigma-Aldrich (Sigma-Aldrich, St. Louis, MO; United States). Stock solutions (100 mg L^−1^) were prepared in methanol and stored at 4 °C. Working solutions were prepared daily by diluting appropriate quantity of stock standard solution with ultrapure water. Acetonitrile (≥99.9%) CHROMANORM® of LC-MS grade was purchased from VWR International (VWR International, Radnor, Pennsylvania, United States).

For the synthesis of the magnetite nanoparticles (Fe_3_O_4_), iron(iii) chloride hexahydrate (FeCl_3_·6H_2_O), iron(ii) chloride tetrahydrate (FeCl_2_·4H_2_O) and ammonia solution (NH_3_) were purchased from Sigma-Aldrich. All chemicals used during the synthesis of the sorbent were of reagent grade. Wood-based activated carbon (BAX-1500) was manufactured by Mead Westvaco (Richmond, Virginia, USA). River and lake surface water samples were collected in Vienna (Austria) and filtered with nylon filter membranes, pore size 0.45 μm, prior to the MSPE procedure.

### Synthesis of magnetite nanoparticles (Bm)

Initially, magnetite nanoparticles were synthesized by the chemical co-precipitation approach according to the modified Massart Method.^23^ In particular, a mixture of FeCl_2_·4H_2_O (1.13 g, 5.6 mmol) and FeCl_3_·6H_2_O (3.03 g, 11.2 mmol) (1 : 2 mass ratio) were dissolved in 150 mL deionized water, followed by heating at 60 °C under vigorous stirring and inert N_2_ atmosphere to obtain a clear yellow solution. Subsequently, aqueous ammonia solution was added dropwise until the pH of the solution reached the value of 10. The suspension was heated and kept at 90 °C for 1 h while N_2_ was used as the protective gas throughout the experiment. The black precipitate was collected by an external magnetic field, and after subsequent washing with water and ethanol freeze–drying took place.^15,24–29^

For the preparation of the magnetite-impregnated activated carbon, the wood-based activated carbon, BAX-1500 (B)^15^ was initially washed in a Soxhlet apparatus to remove soluble impurities. Then, half a gram of the material was dispersed in 150 mL water under ultrasonic irradiation for 30 min, until a homogenous suspension was obtained. Subsequently, magnetite (0.25 g) was added and the mixture was sonicated for 30 min. Finally, the resulting magnetic micro–meso porous activated carbon was collected by a magnet and freeze–dried.

### Instrumentation

For the characterization of the surface structure and morphology of the samples, scanning electron micrographs (SEM) of the prepared composite were taken using a FEI Quanta 200 SEM instrument, (FEI, Hillsboro, OR, USA). The FT-IR spectra of the adsorbents before and after the adsorption of caffeine were obtained by a PerkinElmer FTIR spectrophotometer (model Spectrum 1000) in the range 2000–450 cm^−1^ using KBr pellets (Shelton, CT, USA). The spectra are presented in transmittance mode and were subjected to baseline correction.

A Shimadzu GC-2010 Ultra gas chromatograph with quadrupole mass spectrometry (MS) detector (Shimadzu, Tokyo, Japan) was used for determination of caffeine. Helium (99.999%, Messer, Gumpoldskirchen, Austria) was used as the carrier gas at a constant volumetric flow rate of 1.5 mL min^−1^. The injector temperature was 280 °C, while ion source and interface temperatures were 250 °C and 200 °C, respectively. A DB-5-MS Ultra Inert column (25 m × 0.25 mm internal diameter, 0.25 μm film thickness) was used as stationary phase (Agilent, Santa Clara, CA, USA). The oven temperature program was as follows: initial temperature 100 °C, ramp to 200 °C at 45 °C min^−1^ (held for 7 min), ramp to 320 °C at 45 °C min^−1^ (held for 3 min). Injection of 2 μL of sample was performed with a split ratio of 1 : 10. Solvent delay was set at 4.0 min and the total run time was 14.9 min. The elution time of caffeine was 5.5 min and therefore the mass detection was stopped after 6.5 min. Caffeine was quantified in selected ion monitoring (SIM) mode recording *m*/*z* 194 as the target ion and *m*/*z* 109 for qualitative confirmation.

A VWR Ultrasonic bath was used for the ultra-sound assisted extraction (VWR International, Radnor, Pennsylvania, United States). Moreover, for the preparation of Bm a heating magnetic stirrer (RW 20 digital, IKA Labortechnik, Staufen, Germany) was employed.

### MSPE procedure

For the MSPE procedure, 2.5 mg of the sorbent were placed into a 40 mL glass vial and 20 mL of the water sample was added. Sodium chloride was added to a final concentration of 5% w/v. Dispersion of the magnetic sorbent in the sample solution took place in an ultrasonic bath for 10 min to adsorb the target analytes. Subsequently, the sorbent was separated with the aid of an external magnetic field and the aqueous phase was discarded. For the elution step, an aliquot of 500 μL acetonitrile was added to the magnetic sorbent and the mixture was subjected to ultrasonic irradiation for 2.5 min. Thereafter, the eluent was separated from the magnetic nanocomposite and 2 μL of the acetonitrile phase were injected into the GC-MS.

### Method validation

The developed MSPE method was validated in terms of linearity, limit of detection (LOD) and limit of quantification (LOQ), accuracy and precision. The linearity of the proposed method was evaluated by linear regression analysis. For this purpose, calibration curves were constructed by plotting the peak area of caffeine *versus* the concentration for standard solutions and spiked sample solutions. The slope, intercept, and coefficient of determination were calculated by least square linear regression analysis. The LOQ value of the herein developed method was the lowest point of the calibration curve, while the LOD value was calculated by dividing the LOQ by 3.3. The extraction recovery (R%) of caffeine was calculated by comparing the theoretical concentration and the real concentration after elution. Similarly, enhancement factor (EF) was calculated as the ratio of analyte concentration after elution to the initial concentration of caffeine. The accuracy of the MSPE-GC-MS method was evaluated in terms of bias between nominal and measured concentrations of spiked samples. For the evaluation of the intra-day repeatability, analysis of spiked samples in five replicates in the same day was performed. Accordingly, the relative standard deviation (RSD%) was calculated. Finally, inter-day precision and accuracy were evaluated by analyzing spiked samples thrice in five different days and calculating the RSD%.^7,30,31^

## Results and discussion

### Material characterization and extraction mechanism

**Fig. 1:**
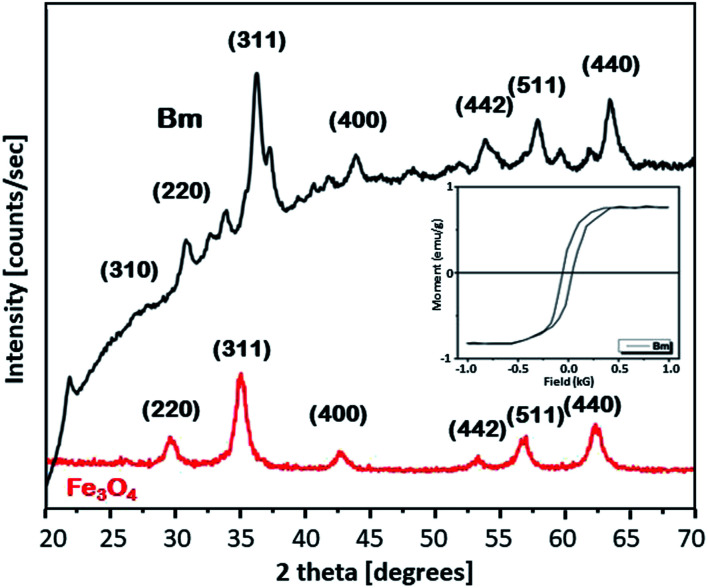
XRD diffractograms for Fe_3_O_4_ and Bm and magnetization curve for Bm (inset figure).

To get further insight into the mechanism of the caffeine interaction with the magnetic activated carbon surface and hence its suitability for the proposed method, the structural and chemical features of the carbon are presented. The magnetic activated carbon examined was previously characterized.^15,25^ The iron content of Bm estimated by chemical analysis^32^ was found to be 28.77%, while the identification of the particular iron phase (or: oxide) was performed by X-ray diffraction and the diffractometer exhibited diffraction peaks corresponding to (1 1 1), (2 2 0), (3 1 1), (4 0 0), (5 1 1) and (4 4 0) planes of the cubic crystal structure (fcc) of magnetite (JCPDS file no. 19-0629),^26^ consistent with the diffraction peaks of the pure magnetite, also presented in Fig. 1. The average crystallite size *D* of the particles impregnated in the activated carbon, estimated from the Debye–Scherrer equation, was found to be 12.3 nm. The magnetic properties of the Bm carbon, measured with vibrating sample magnetometry (VSM) at room temperature and presented in the inset of Fig. 1, revealed superparamagnetic characteristics with a saturation magnetization of *σ*_s_ = 18 emu g^−1^, indicating that the magnetic particles are magnetically single domain and the magnetic carbon was adequate for magnetic separation.

Nitrogen adsorption–desorption isotherms for the raw and Bm activated carbon as well as for magnetite are presented in Fig. S1 (ESI†). From the isotherms it can be evaluated that B carbon can be considered as micro–meso-porous with a surface area of 2490 m^2^ g^−1^ and total pore volume of 1.619 cm^3^ g^−1^ while after magnetite impregnation, the surface area for Bm carbon decreases to 1559.8 m^2^ g^−1^ and the total pore volume to 1.170 cm^3^ g^−1^ mainly due to pore blocking effects as a result of magnetite deposition. Table S1 (ESI†) summarizes the textural parameters of Fe_3_O_4_, B and Bm. Pore size distributions curves (PSDs) are presented in Fig. S2† and reveal that after magnetite impregnation, a decrease in the pore volume for pores larger than 20 Å possibly occurred due to the deposition of magnetite in larger pores, as well as a slight increase in the volume of pores smaller than 10 Å possibly due to the formation of new pores attributed to the magnetite impregnation. The results indicate that activated carbon is a suitable adsorbent for caffeine with a molecular diameter of about 0.78 nm.

**Fig. 2:**
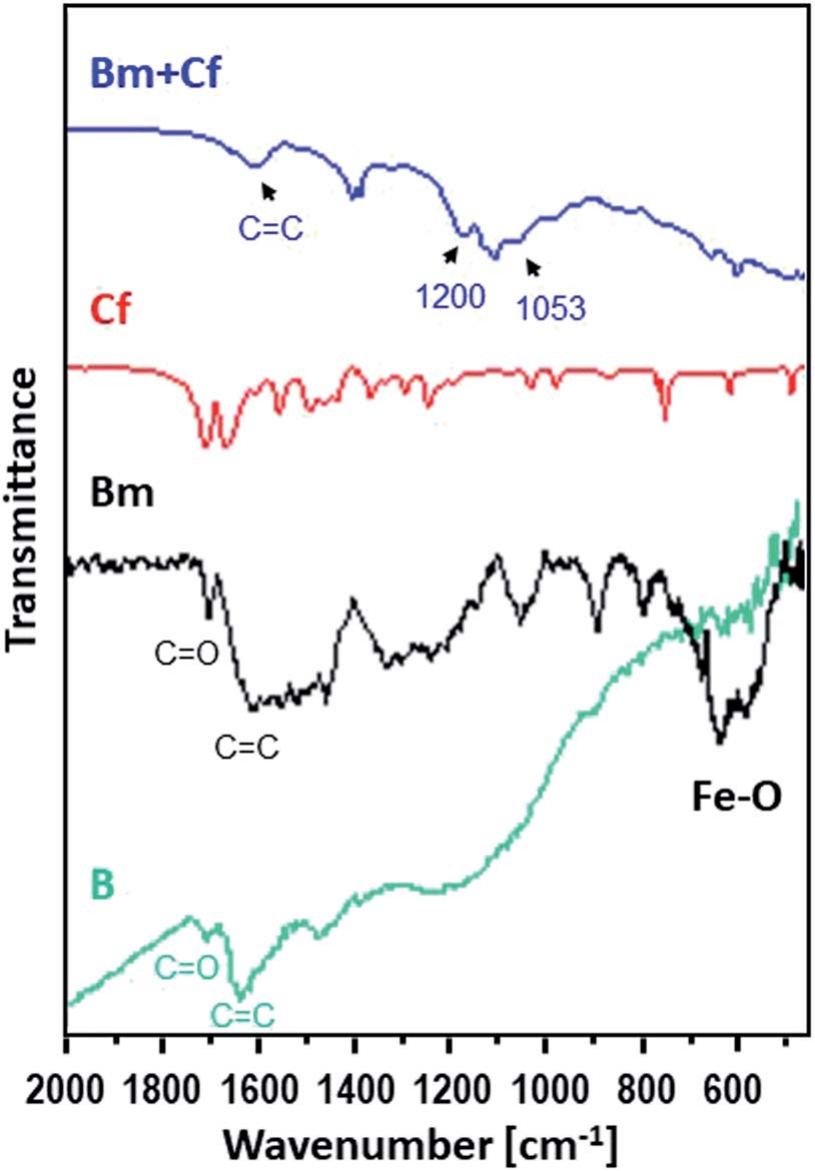
FTIR spectra for caffeine (red), for the activated carbon sorbent B (green) and for Bm before (black) and after (blue) the adsorption of caffeine.

Moreover, Fig. 2 shows the FTIR spectra of the raw activated carbon, B, and of the magnetic carbon, Bm, as well as its counterpart after caffeine adsorption, providing information about their surface chemistry and the interactions of caffeine with the surface of magnetic activated carbon.

The main characteristic absorption peaks of B sample could be assigned at 1700 cm^−1^ to the stretching vibration of C=O in carboxyl groups, at 1630 cm^−1^ to the aromatic skeletal stretching (C=C) vibration in aromatic rings, at 1460 cm^−1^ to vibrations of the C–C group and in the range of 1000–1300 cm^−1^ to the vibrations of the functional groups of –CO and –OH. For the magnetic Bm carbon, the band at 1700 cm^−1^ is assigned to the carboxyl C=O stretching while the band at about 1620 cm^−1^ is the combination of C=C stretching vibration of the aromatic ring structures and conjugated systems such as diketones, ketoesters, quinones. The bands at 1580 and 1400 cm^−1^ are assigned to the asymmetric and symmetric carboxylate units coordinated to the magnetite while the bands from ∼1300 cm^−1^ to ∼1000 cm^−1^ are assigned to C–O–C lactone structures, stretching C–O vibrations of phenol structures and ethers and bending O–H modes of phenol structures and at ∼900 cm^−1^ to epoxy groups.^33^ These bands presented a higher intensity indicating that surface oxygen groups would facilitate the attachment of magnetic nanoparticles through a covalent coupling or electrostatic interactions. The bands at 695.2 and 468.8 cm^−1^ correspond to the Fe–O bond vibration of impregnated Fe_3_O_4_ nanoparticles.^34,35^

The FTIR spectrum of magnetic activated carbon, after caffeine adsorption, also presented in Fig. 2, presents evidence about the adsorption of caffeine onto the carbon surface as well as about the adsorption mechanism. The spectrum of magnetic activated carbon, after caffeine adsorption presents a shift of the band due to C=C stretching vibration of the aromatic ring structures, compared to pure magnetic carbon, from 1683 to 1607 cm^−1^, due to π–π electron donor–acceptor interaction, also known as π–π interaction. The interaction exists between the π electron-rich surface of magnetic carbon and the π electron cloud of the two aromatic rings of caffeine.^36^ Besides, the n–π electron–donor–acceptor interaction, also known as n–π interaction, which occurs between lone pair electron-rich atoms such as oxygen on the surface of carbon and π electron cloud of caffeine molecules as was also discussed in Tran *et al.*^37^ The broadening of peak intensity of C=C, at 1657, as well as the band at 1053 cm^−1^ and the band at ∼1200 cm^−1^ due to C–N supports the assumption of caffeine adsorption on the surface of carbon.^38^ Besides, in the pH region between 4 and 10.4, caffeine exists in its cationic form and since all experiments were performed at pH ∼ 7, the carbon surface was negatively charged due to the predominance of the negatively charged carboxylate moieties. Electrostatic attraction could be also developed between the adsorbate and the adsorbent, indicating that the adsorption of caffeine on the magnetic carbon surface could be also be based on electrostatic interactions.^39^ Additionally, the appearance of C–N stretching vibration at ∼1200 cm^−1^ in the caffeine treated carbon demonstrated that the caffeine donated electrons to the carbon surface, while the intensity increase of the C–O absorption at ∼1053 cm^−1^ showed that the caffeine acted as an electron acceptor from the carbon. Thus, caffeine was also adsorbed on magnetic carbon surface *via* the electron donor–acceptor mechanism. The N atom attached in the five membered (imidazole) ring of caffeine can be considered as electron donor to the C=O group attached to the carbon surface, whereas the C=O group in the six membered (pyrimidine) ring of caffeine can act as electron acceptor.^40,41^ From FTIR results it is seen that caffeine can be successfully adsorbed on magnetic carbon's surface.

The SEM micrographs of Bm activated carbon, presented in Fig. S3,† show that the surface texture presents a spongy structure that exhibits defects and cavities possibly attributed to the impregnation of Fe_3_O_4_ nanoparticles. Moreover, no significant changes were observed on the material's surface, after the adsorption of caffeine.

### Optimization of MSPE conditions

#### Optimization of adsorption step

For the optimization of the adsorption step the following parameters were evaluated: adsorption time, sample volume and salt addition. For the sample preparation of 20 mL of sample, different quantities (2.5, 5, 10, 15 and 20 mg) of the micro–meso porous activated carbon/Fe_3_O_4_ nanocomposite were evaluated. It was found that 2.5 mg of the sorbent was sufficient to adsorb the caffeine. Further increase of sorbent quantity did not lead in any increase of the extraction efficiency. Therefore, further experiments were conducted with 2.5 mg of micro–meso porous activated carbon/Fe_3_O_4_ nanocomposite as adsorbent.

**Fig. 3:**
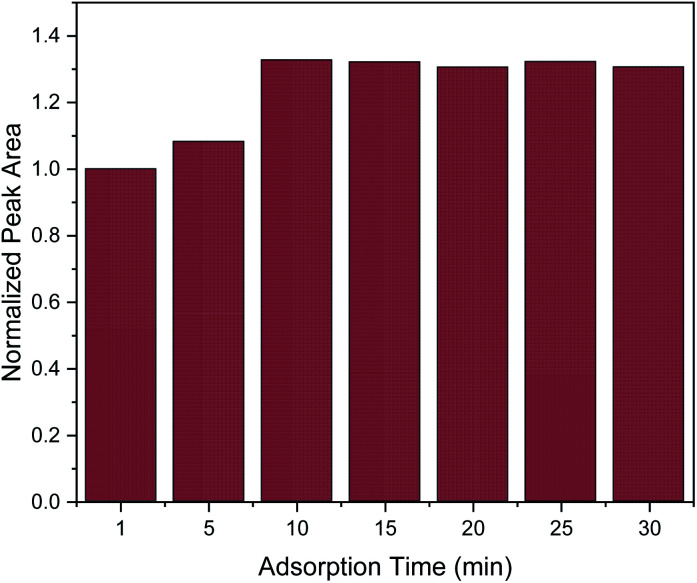
Effect of adsorption time on the MSPE process. Normalization of peak area was performed by dividing the peak area for each value by the peak area at 1 min.

In order to obtain the maximum extraction efficiency, sufficient adsorption time is required for the complete dispersion of the sorbent in the sample, as well as for effective mass transfer in order to reach the extraction equilibrium.^2,3^ Herein, adsorption time of 1, 5, 10, 15, 20, 25 and 30 min were evaluated. As shown in Fig. 3, the extraction efficiency increased from 1 to 10 min and no further increase was observed afterwards. Therefore, 10 min was chosen as the optimum adsorption time.

**Fig. 4:**
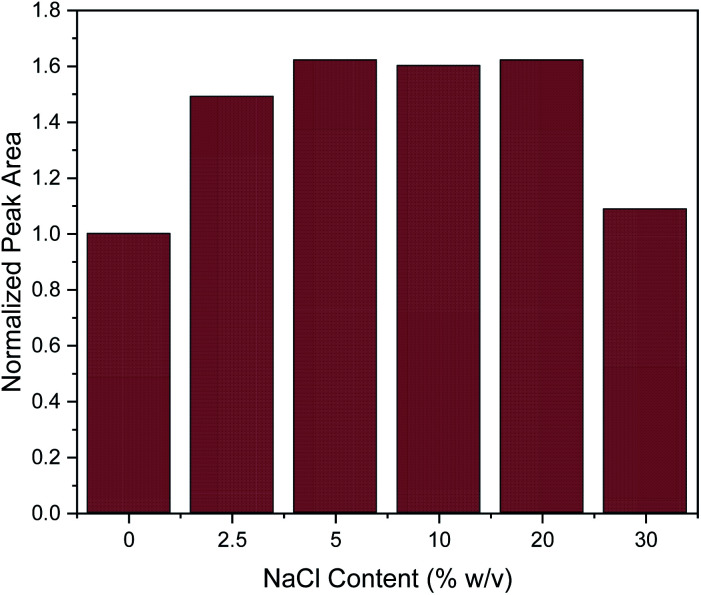
Effect of salt addition on the MSPE process. Normalization of peak area was performed by dividing the peak area for each value by the peak area of 0% w/v.

Subsequently, the addition of NaCl was evaluated at different concentrations (0, 2.5, 5, 10, 20 and 30% w/v). The salting-out effect can enhance the extraction efficiency, by decreasing the solubility of organic analytes.^3^ This was also the case here at the lower of the investigated salt concentrations, since the addition of NaCl up to the concentration of 5% w/v enhanced the extraction efficiency of caffeine, as shown in Fig. 4. Then, the extraction efficiency remained constant until the concentration of 20% w/v. However, further increase in NaCl concentration led to a drastic decrease of extraction efficiency. This can be explained by the significant increase in viscosity of the solution at high slat concentrations, resulting in a hindrance of the adsorption of the target analytes by reducing the extraction efficiency and diffusion coefficient.^3^ This explanation is supported by data published for the dynamic viscosity *η* of aqueous NaCl solutions: for an aqueous solution of 5% w/v NaCl, *η* = 0.89 mPa.s and for 20% w/v NaCl, *η* = 1.12 mPa.s while for a 30% w/v NaCl solution this value increases to *η* = 1.57 mPa.s (data interpolated from Zhang and Han^48^). This represents a 40% increase in viscosity which may well explain the decrease of extraction efficiency of approximately the same order of magnitude. At the lower concentrations of NaCl, the increase in solvent viscosity is comparatively small, and more than compensated by the above-mentioned salting-out effect. Therefore, all further experiments were conducted with a salt addition of 5% w/v.

#### Optimization of elution step

For the optimization of the elution step the following parameters were examined: type of eluent, desorption time and volume of desorption solution. In order to avoid incomplete desorption of the analyte, which can lead to carry-over effect and decreased extraction efficiency, it is important to find the optimum desorption conditions.

**Fig. 5:**
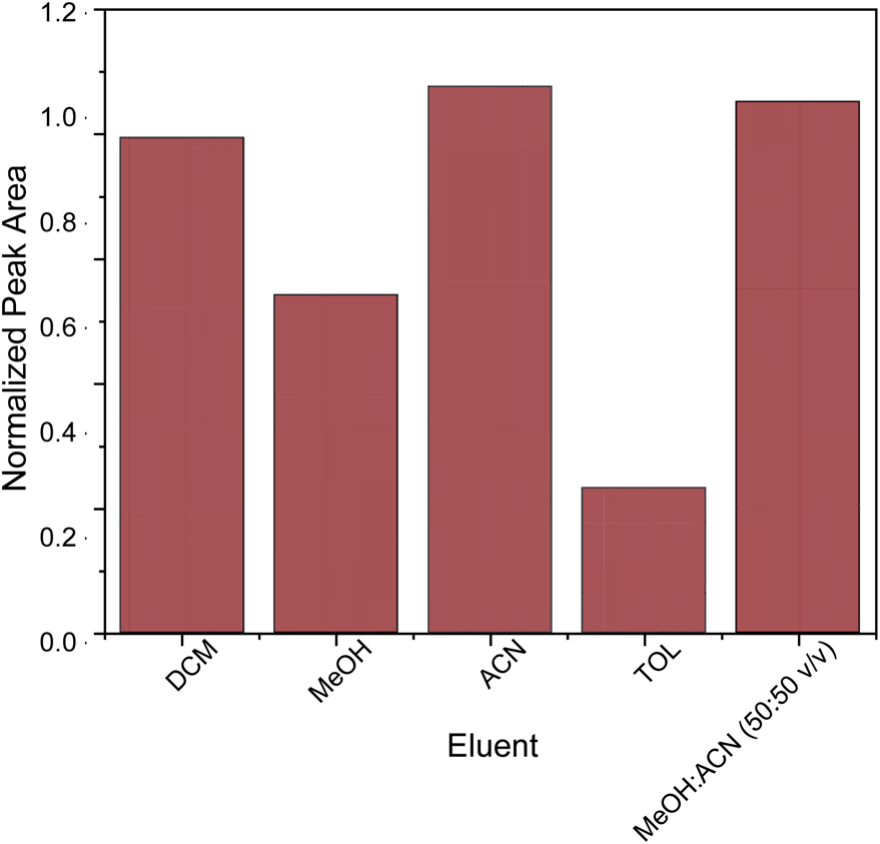
Optimization of eluent.

The key factor for the selection of the most appropriate elution solvent is polarity.^42^ For this reason, elution solvents of different polarities were evaluated. The studied elution solvents were acetonitrile (ACN), methanol (MeOH), toluene (TOL), dichloromethane (DCM) and a mixture of MeOH : ACN 50 : 50 v/v. As shown in Fig. 5, acetonitrile and the mixture of MeOH : ACN 50 : 50 v/v exhibited the strongest desorption power among the examined eluents. In order to avoid the need for solvent mixing, pure acetonitrile was chosen as elution solvent. Subsequently, different quantities of acetonitrile (100, 250, 500 and 1000 μL) were evaluated. Generally, the quantity of the elution solvent should be sufficient for the complete elution of the adsorbed analyte from the sorbent. It was found that 500 μL of acetonitrile was sufficient for the elution of caffeine from the micro–meso porous activated carbon/Fe_3_O_4_ nanocomposite.

**Fig. 6:**
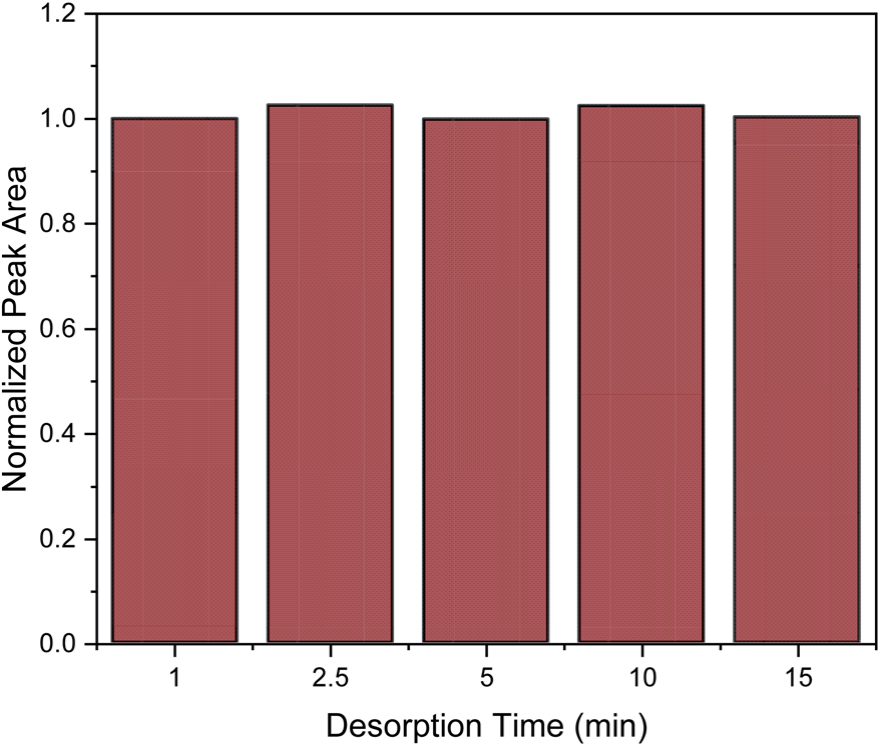
Effect of desorption time on the MSPE process. Normalization of peak area was performed by dividing the peak area for each value by the peak area of 1 min.

Desorption time is another critical factor that should be carefully optimized in order to provide complete elution of the target analytes from the nanocomposite. For the evaluation of desorption time the following desorption time spans were studied: 1, 2.5 5, 10 and 15 min. As it can be observed in Fig. 6, one minute was enough to desorb the target analyte. However, for reproducibility reasons, a desorption time of 2.5 min was chosen.

Normalization of peak area was performed by dividing the peak area for each value by the peak area of DCM.

### Analytical performance of the MSPE-GC-MS method

The analytical performance results including the calibration function, the coefficient of determination, the LOD and LOQ values, the extraction recoveries and the enrichment factors of the developed MSPE-GC-MS are listed in Table 1. As it can be seen, a wide linear range was obtained, and the coefficient of determination was 0.9992 indicating good linearity for caffeine. Moreover, the LOD and LOQ values were found to be 0.18 and 0.60 ng mL^−1^, respectively. Under the optimum conditions, extraction recovery was 91.1% and enhancement factor was 36.4, close to the theoretical value of 40, calculated from the volume ratio. The relative standard deviation for intra-day values (*n* = 5 replicates, *c* = 25 ng mL^−1^) was 5.6% and for the inter-day values (*n* = 5 days *×* 3 replicates, *c* = 25 ng mL^−1^) was 6.2%.

**Table 1:** Validation results for the developed MSPE-GC-MS method

Validation parameter	Result
Calibration curve (*y* = peak area, *x* = ng L^−1^)	*y* = 2233.1.1*x* + 1067.7
*R* ^2^	0.9997
Linear range (ng mL^−1^)	0.60–12.5
LOD (ng mL^−1^)	0.18
LOQ (ng mL^−1^)	0.60
Extraction recovery (%)	91.1
Enhancement factor	36.4
Intra-day RSD%	5.6
Inter-day RSD%	6.2

### Real samples analysis

The MSPE-GC-MS procedure was applied in the analysis of surface water samples. No caffeine was detected in thesamples. The relative recovery (RR%) of caffeine was studied by spiking the caffeine standard solution into the samples (*c* = 6.0 ng mL^−1^). As seen in Table S2,† the relative recoveries for caffeine in real water samples were in the range from 93.3 to 96.7%. The absence of interferences in the spiked samples and the clean background signal, in combination with the satisfactory relative recovery values indicated absence of matrix effect for the proposed MSPE-GC-MS method. The developed MSPE-GC-MS method was compared with other solid-phase extraction methods reported in the literature for the determination of caffeine, as shown in Table 2. These have been used in combination with various detection systems including HPLC-UV,^43,44^ GC-MS^45,46^ and electrospray ionisation ion mobility spectrometry (ESI-IMS).^47^ It is remarkable that only 2.5 mg of Bm was required for the sample preparation process, while in most research papers the quantity of the magnetic sorbent ranged between 15–100 mg.^43–45,47^ Adsorption and desorption time were lower than those reported in ref. 43,46 and 47 but higher than those reported in ref. 45. The adsorption and desorption time in ref. 47 was calculated based on the flow rate (0.03 mL min^−1^) as well as the sample and eluent volumes, respectively.

**Table 2:** Comparison of the developed MSPE-GC-MS method with other methods for the quantitative analysis of caffeine

Sorbent	Sample preparation technique	Detection system	Sorbent mass (mg)	Adsorption time (min)	Desorption time (min)	Relative standard deviation (%)	Linear range (ng mL^−1^)	LOD (ng mL^−1^)	Ref.
Graphene oxide	Ultra-sound assisted d-SPE	HPLC-UV	15	15	5	1.8 (intra-day) 2.9 (inter-day)	(0.003–5) × 10^3^	0.11	43
Octadecyl silica	On-line SPE	HPLC-UV	100	—	—	>12% (intra-day)	(15–200) × 10^−3^	0.01	44
3D-graphene	Ultra-sound assisted MSPE	GC-MS	50	0.5	0.5	5.9 (intra-day) 7.1 (inter-day)	(0.5–500) × 10^3^	100	45
Molecular sol–gel imprinted fiber	SPME	GC-MS	—	60	—	10 (intra-day) 16 (inter-day)	(1–80) × 10^3^	100	46
Molecularly imprinted polymer	SPE	ESI-IMS	50	>15	>15	<6	(0.5–20.00) × 10^3^	200	47
Bm	Ultra-sound assisted MSPE	GC-MS	2.5	10	2.5	5.6 (intra-day) 6.2 (inter-day)	0.60–12.5	0.18	This work

The RSD% of the proposed method was similar to those of ref. 45 and 47 and it can be considered as satisfactory, while it was better compared to that of the on-line SPE method (ref. 44). Finally, it should be noted that the LOD of this study was similar to the LOD value reported in ref. 43, higher that the LOD value reported in ref. 44, but lower than the LOD value of ref. 45–47.

## Conclusions

A rapid, simple, economic and sensitive method was developed using micro–meso porous activated carbon/Fe_3_O_4_ nanocomposite as adsorbent for the magnetic solid-phase extraction of caffeine from surface water samples prior to their determination by GC-MS. The novel sorbent exhibited sufficient magnetic properties, as well as high enhancement factors and extraction recoveries. The herein developed MSPE method showed a wide linear range, as well as and satisfactory intra-day and inter-day repeatability. Among the limitations of the proposed method is that sorbent reusability was not considered, since its collection after the MSPE process without losses was not possible. However, in order to extract the target analytes from real samples, only a small quantity of sorbent was required (2.5 mg). A significant benefit of the proposed sample preparation technique is the simplicity and low cost of the synthesis of Bm sorbent. Moreover, since only a small quantity of organic solvent is required, the proposed MSPE procedure is considered environmentally friendly. Finally, it is important to mention that although only lake and river surface waters were analyzed in this study, this method is also likely to be applicable to sea surface water samples, since a salt addition of 5% w/v NaCl was found to be beneficial for the extraction. Moreover, the applicability of this method could be potentially expanded (after appropriate modification) for the analysis of food samples and pharmaceutical products that may contain caffeine.

## Author contributions

N. M. – conceptualization, investigation, writing – original draft; E. A. D. – conceptualization, resources, investigation, writing – review & editing; E. R. – conceptualization, resources, supervision, validation, writing – review & editing; G. A. Z. –conceptualization, supervision, writing – review & editing.

## Conflicts of interest

There are no conflicts to declare.

## Supplementary Material

RA-011-D1RA01564H-s001
