# Supplementary material for: Magnetic solid-phase extraction of caffeine from surface water samples with a micro–meso porous activated carbon/Fe3O4 nanocomposite prior to its determination by GC-MS
Source: RSC Adv. 2021 May 28;11(32):19492–9. doi: 10.1039/d1ra01564h (PMC9033622; doi:10.1039/d1ra01564h)
Supplement: RA-011-D1RA01564H-s001 [file RA-011-D1RA01564H-s001.pdf]

Electronic Supplementary Material (ESI) for RSC Advances.
This journal is © The Royal Society of Chemistry 2021

SUPPLEMENTARY MATERIAL

Magnetic solid-phase extraction of caffeine from surface water samples with micro-meso porous activated carbon/Fe_3O_4 nanocomposite prior to its determination by GC-MS

N. Manousi[a], E. A. Deliyanni[b], E. Rosenberg[*c], G. A. Zachariadis[a],

[a] *Laboratory of Analytical Chemistry, Department of Chemistry, Aristotle University of Thessaloniki, Thessaloniki 54124, Greece*

[b] *Laboratory of Chemical and Environmental Technology, Department of Chemistry, Aristotle University of Thessaloniki, Thessaloniki 54124, Greece*

[c] *Institute of Chemical Technology and Analytics, Vienna University of Technology, 1060 Vienna, Austria*

Table S1. Textural parameters of Fe_3O_4, B and Bm

Adsorbent	SSA m^2/g	V_{tot} (cm^3/g)	V_{mic} (cm^3/g)	V_{mes} (cm^3/g)
**Fe_3O_4**	106.6	0.404	0	0.404
**B**	2490	1.619	0.293	1.326
**Bm**	1559.8	1.170	0.297	0.873

Table S2. Results of the analysis of caffeine in real surface water samples

	River Surface Water Sample			Lake Surface Water Sample		
Analyte	Added (ng mL^{-1})	Found (ng mL^{-1})	RR %	Added (ng mL^{-1})	Found (ng mL^{-1})	RR %
Caffeine	0	ND	-	0	ND	-
	6.0	5.8 ± 0.6	96.7	60.0	5.6 ± 0.5	93.3

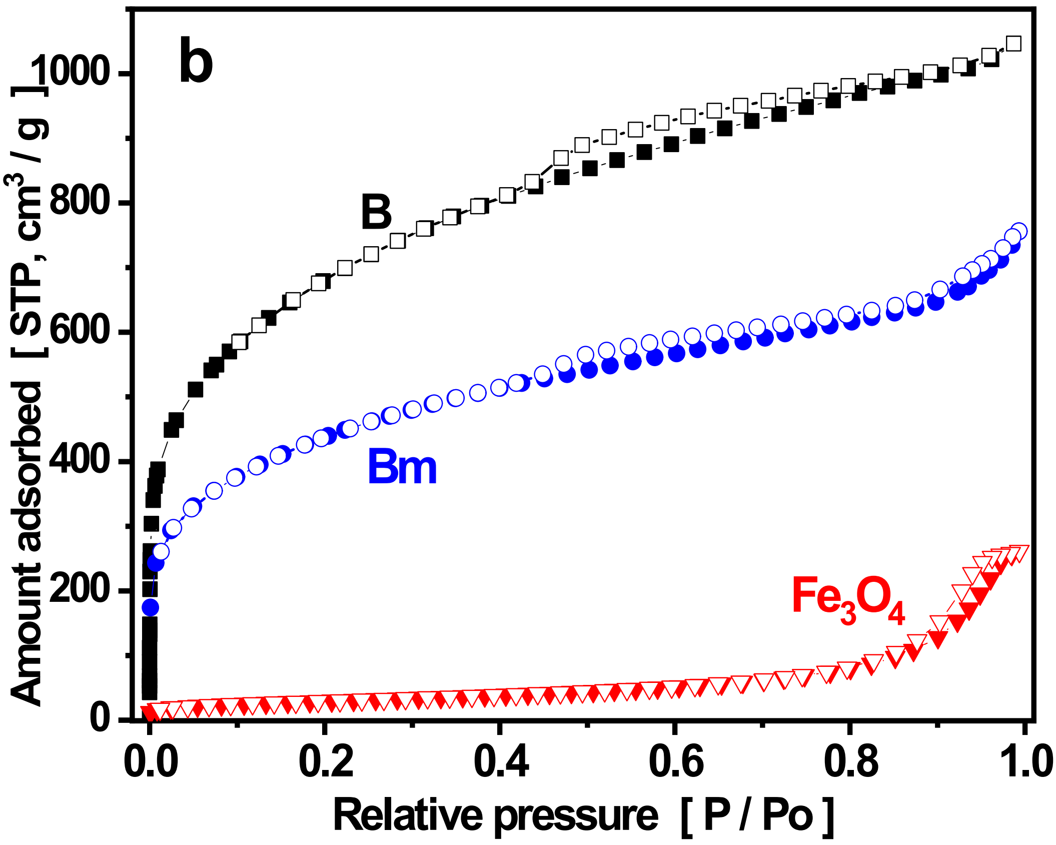

Figure S1. N_2 adsorption (open symbols)–desorption (closed symbols) isotherms for Bm

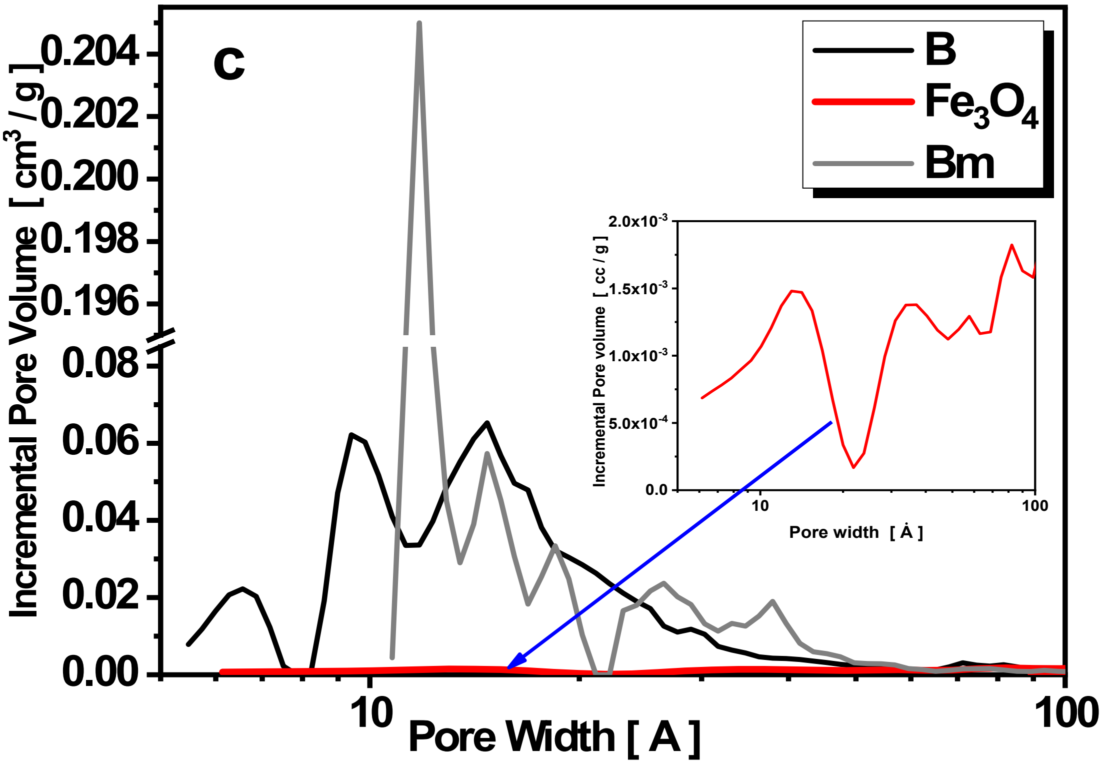

Figure S2. Pore size distribution for Bm

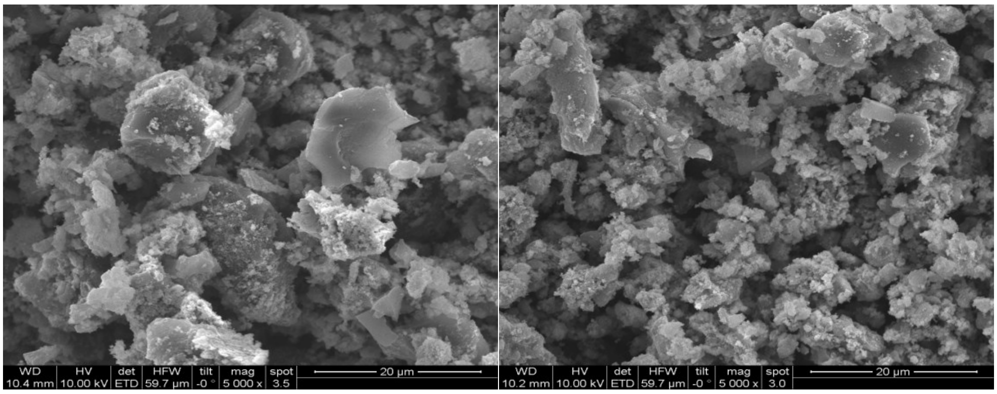

Figure S3. SEM micrographs of Bm activated carbon before (left) and after (right) the adsorption of caffeine
